# Efficacy of a Web-Based Integrated Growth Mindset Intervention on Reducing Anxiety Among Social Work and Counseling Practicum Trainees: Protocol for a 2-Arm Randomized Controlled Trial

**DOI:** 10.2196/67234

**Published:** 2025-03-27

**Authors:** Yongyi Wang, An Xi, Stella S K Wong, Kong Yam, Janet Tsin Yee Leung, Shimin Zhu

**Affiliations:** 1 Department of Applied Social Sciences The Hong Kong Polytechnic University Hong Kong China (Hong Kong); 2 Mental Health Research Centre The Hong Kong Polytechnic University Hong Kong China (Hong Kong)

**Keywords:** implicit theory, growth mindset, social work students, counselling students, practicum, anxiety

## Abstract

**Background:**

Practicum is indispensable for the development of professional practitioners; yet, trainees may encounter psychological distress, especially anxiety, brought on by new challenges. Research stated that a positive mindset promotes better learning and mental status. Well-designed interventions have been shown to relieve anxiety and help trainees thrive in their practicums and professions. The proposed study adapted an integrated intervention, We-SMILE (Web-Based Single-Session Intervention of Mindset on Intelligence, Failure, and Emotion), for improving prepracticum anxiety and coping. We-SMILE has the potential to be a low-intensity self-help prepracticum intervention to support students in adjusting their mindsets and overcoming the challenges in practicum.

**Objective:**

Using a 2-arm randomized controlled trial, this study aims to examine the efficacy of We-SMILE on reducing anxiety (primary outcome) and enhancing psychological status, psychological well-being, learning orientation, academic self-efficacy, and confidence (secondary outcomes).

**Methods:**

A total of 117 students will be recruited from the social work and counseling programs and randomly assigned to existing prepracticum training (training as usual [TAU]) or that plus the We-SMILE. Participants will be assessed repeatedly at 3 time points: baseline, 2 weeks post intervention, and 8 weeks post intervention. The outcomes will be measured by validated items and scales on anxiety, mindsets, psychological well-being, and the Failure Mindset Scale. Recruitment for the pilot study was initiated in May 2024 during social work and counseling prepracticum briefing sessions. Participants were randomly assigned to the intervention or TAU group. The intention-to-treat (ITT) analysis principle and linear regression–based maximum likelihood multilevel models will be used for data analysis.

**Results:**

This study has received research ethics approval in May 2024. Participant recruitment started at the end of May 2024, and enrollment was ongoing as of when this protocol was submitted. Data collection and analyses are expected to be complete in 2025.

**Conclusions:**

The randomized controlled trial will compare the efficacy of the We-SMILE intervention group and the TAU group. The results of this study will benefit practicum students, fieldwork supervisors, and social work and counseling programs.

**Trial Registration:**

ClinicalTrials.gov NCT06509802; https://tinyurl.com/36vkwd63

**International Registered Report Identifier (IRRID):**

DERR1-10.2196/67234

## Introduction

### Prepracticum Anxiety

Practicum is an essential educational component of professional training [1,2], bridging the gap between theory and practice and enhancing the professional capacity and core competence of future practitioners [3,4]. The quality of fieldwork profoundly influences social work trainees’ personal, intellectual, and professional development [4], per communication skills, critical reflection, professional growth, creativity, innovation, and self-efficacy [1,5]. Students and alumni frequently highlight their field experiences as pivotal in preparing them for their future roles [1]. Research indicated that continuous improvements in fieldwork training benefit the training of future practitioners, such as integration of resources, collaboration across sectors, and improvements in curriculum design [1,4,5]. Through practice and feedback, trainees gradually evolve from knowledge receivers to social workers and competent practitioners [6]. However, the process can be challenging and sometimes frustrating, with obstacles such as the rigorous training process and mental distress [4].

Research showed that students indeed face various challenges during practicums, which can be categorized into professional and individual levels [7]. At the professional level, anxiety and stress may rise when students experience issues with engagement, poor relationships, etc, related to trainers and supervisors [1,2,8,9] and are often exacerbated by the distinct cultural context and intense competition [6,7]. Moreover, although the programs usually prepare students for their placements, they may overlook the specific complexities of the work environment and the higher expectations of professional skills [4,10]. On the flip side, students probably encounter mental difficulties (eg, anxiety, stress, and compassion fatigue), financial pressures, maladaptive coping, physical problems, etc [7,11-15]. It has been noted that excessive anxiety and negative emotions can interfere with the practicum process [16]. Being adequately prepared to confront the challenges and difficulties in practicum is crucial for the learning outcomes and well-being of social work and counseling trainees.

Different coping mechanisms among students can lead to varied training outcomes. Clinical practicum students with the abilities to both manage their emotions and understand the emotions of the people around them have been shown to achieve better patient outcomes and patient satisfaction [2]. For example, dietitian students who effectively manage stress report the most supported feeling [10]. Conversely, students may doubt their abilities and talents if they find it tough to handle critical feedback and expectations from their supervisors and placement agency staff, besides the discrepancy between their preconceived notions before the practicum and the real situation [4,9]. Thus, fostering a positive attitude toward negative emotions, challenges, and feedback is essential to facilitate better learning outcomes and maintain trainees’ mental health.

### Existing Interventions and Gaps

The existing intervention approaches have broadly been delivered from 2 perspectives: the external perspective, such as transition support programs and peer group supervision [17-19], and the internal perspective, such as mindfulness training [20]. Transition support programs usually focus on supervisory support, transition-supportive learning activities, professional behavior and practice, and student internship responsibilities [4,18]. These programs are beneficial to students’ mental health, specifically lowering anxiety, increasing confidence, improving preparation levels, and enhancing professional knowledge and skills [4,17]. Moreover, researchers examined the efficacy of peer group supervision in a practicum setting for counseling students [19] and found it was helpful to stimulate and bolster participants’ professional self-efficacy, self-confidence, and feelings of pleasure and happiness [21]. However, several pertinent studies particularly highlighted mindfulness interventions for social work and counseling practicum trainees [22-24]. Mindfulness as a self-care practice was valuable for managing trainees’ anxiety and strengthening self-care [20,25].

There is a notable lack of research evaluating the implementation of stress and anxiety management interventions in practicum settings for social work and counseling trainees. First, many studies usually develop and apply multiple-session interventions and must be led by professionals, expanding the trainers’ workload [26]. More importantly, full-time employees and interns generally face different work tasks, challenges, and pressures [27]. However, existing interventions lack clear definitions and distinctions, so more evidence from interns’ perspectives is needed. Lastly, the current interventions require more objective and reliable outcome indicators and well-designed randomized controlled trials. Thus, a low-intensity self-help prepracticum intervention to increase the preparation levels of social work and counseling trainees is desired.

### Mindset Intervention

Mindset, which refers to implicit theory, means an individual’s belief in the changeability of his or her attributes [28]. Individuals with growth mindsets believe that their attributes are changeable. Believing intelligence and emotion are temporary and evolving will prompt one to make efforts at learning and emotion regulation. In contrast, a fixed mindset indicates the belief that one’s attributes are immutable [29-31]. The extant literature shows that the fixed mindset is related to more anxiety and stress, while the growth mindset contributes to proactive coping with anxiety and stress and resilience in the face of drawbacks [28,32]. Mindset is found to be a modifiable factor in intervention, which is essential in clinical psychology, therapy, prevention, and early intervention. These days, the growth mindset has been gradually introduced into practice and has yielded positive results [31,33,34].

Nevertheless, little research has been conducted on integrated mindset interventions, and there is very limited evidence for this initiative. As mindsets can be domain-specific, one may have different mindsets regarding various domains [35]. When one faces a challenge, multiple mindsets may interact and intervene in one’s coping behaviors. As for prepracticum trainees, their anxiety is multifaceted. Instilling growth mindsets regarding intelligence, emotion, and failure-is-enhancing mindsets, respectively, is worthwhile in easing anxiety and stress coping [36]. An integrated mindset intervention may be more efficient in reducing anxiety related to practicum, thereby preparing for the challenges that may be encountered.

### Integrated Mindset Intervention: We-SMILE

This research has adapted an existing integrated mindset intervention by the principal investigator (PI: SZ), that is, PC-SMILE (Parent-Child Single-Session Mindset Intervention on Intelligence, Failure, and Emotion) for secondary school students [37]. The design is grounded in implicit theory research and supported by emerging evidence of the effect of brief interventions [38-42]. PC-SMILE is a 45-minute intervention that aims to instill growth mindsets of intelligence, failure-is-enhancing, and belief-in-change of emotion using neuroplasticity and real-life examples among students. The efficacy of PC-SMILE is being examined with a 3-arm randomized controlled trial [37].

The current protocol endeavors to modify the child-version of PC-SMILE for practicum trainees in social work and counseling programs, namely, We-SMILE (Web-Based Single-Session Intervention of Mindset on Intelligence, Failure, and Emotion). We-SMILE adheres to the core concepts of PC-SMILE with specific adjustments to practicum trainees’ circumstances, that is, intending to introduce growth mindsets about intelligence, failure, and negative emotion to prepracticum students. We-SMILE starts with stories mirroring scenarios from social work and counseling practicum, followed by key principles from implicit theory, learning mechanisms, and emotion regulation, and finally supports these conceptions with solid evidence from research. One highlight of We-SMILE is that it includes a session dedicated to time management, an issue that new practicum students often struggle with. In this section, students are introduced to effective time management tools and ways of enhancing awareness of allocating time without burning out. Then, transferred to self-care methods to manage emotions caused by practicum experiences.

### Aims

The protocol aims to assess the efficacy of We-SMILE through a 2-arm randomized controlled trial.

The primary objective is to evaluate the efficacy of We-SMILE in reducing anxiety related to practicum among social work and counseling trainees compared to the training as usual (TAU) group.

The secondary objective is to evaluate the efficacy of We-SMILE on secondary outcomes, including (1) relieving depression, anxiety, and stress; (2) improving psychological well-being; (3) enhancing learning orientation; and (4) increasing academic self-efficacy and confidence related to practicum compared with the TAU group.

## Methods

### Design of We-SMILE and Implementation Strategies

Patient and public involvement is a key principle we adopted in the intervention design and implementation strategies. The intervention design was coproduced by the research team and student advisory group who have completed at least one practicum training.

The first step is preintervention development. The needs for prepracticum mindset training were identified through interviews with the students who completed their summer practicum in 2023. Second, during intervention development, a student advisory group of 3 social work students was invited to participate in pilot studies and provide suggestions on the initial questionnaires, videos, and the final intervention regarding content consistency, process clarity, ease of understanding, and intervention duration. Third, after the pilot study in May 2024, participants’ open-ended feedback will be collected for intervention improvement. We also invited 3 participants to interview for detailed comments. Based on the feedback and comments, further improvements were made. Thematic analysis will be conducted to integrate participants’ feedback to identify what they perceive as the most beneficial aspects of the intervention and those most in need of improvement. Coproduction is helpful to ensure the intervention’s acceptability, feasibility, relevance, and effectiveness. Fourth, about implementation strategies. The implementation process was co-designed with the fieldwork coordinators. We collected feedback from supervisors and teachers in the social work program. They will help send invitations to upcoming prepracticum social work and counseling students during briefing sessions and share the research link with the students via WhatsApp group once this study begins. Our study adheres to the CONSORT-EHEALTH (Consolidated Standards of Reporting Trials of Electronic and Mobile Health Applications and Online Telehealth) checklist (version 1.6) and follows the SPIRIT (Standard Protocol Items: Recommendations for Interventional Trials) guidelines (see Multimedia Appendices 1 [43] and 2 for details).

**Figure 1:**
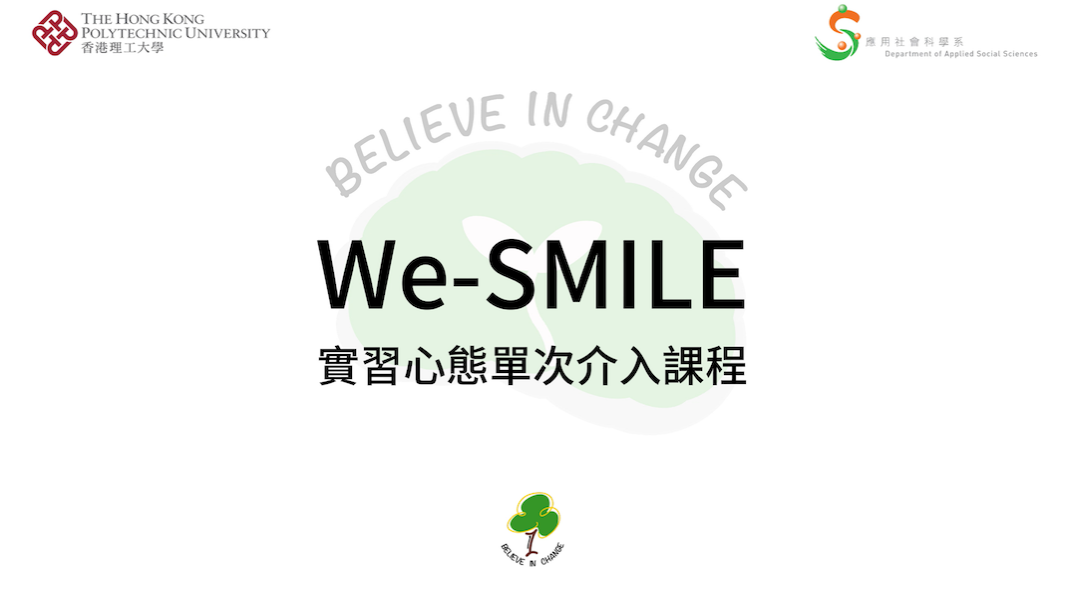
Home page of We-SMILE. We-SMILE: Web-Based Single-Session Intervention of Mindset on Intelligence, Failure, and Emotion.

**Figure 2:**
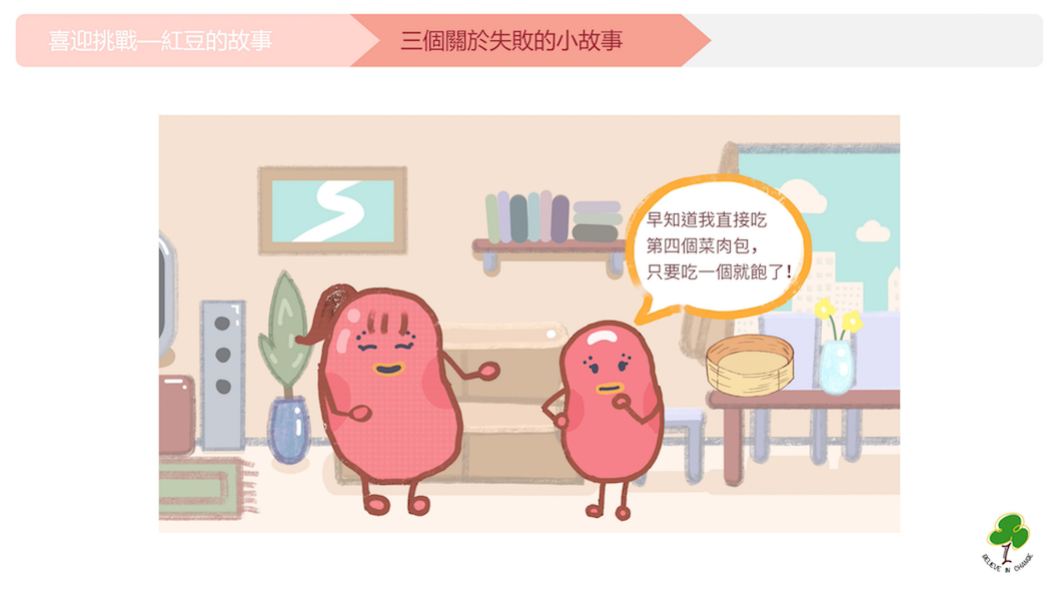
A screenshot from one of three short stories about failure. In the screenshot, Red Bean says, “If only I’d eaten the fourth vegetable and meat bao straight away, just one would have filled me up!”.

**Figure 3:**
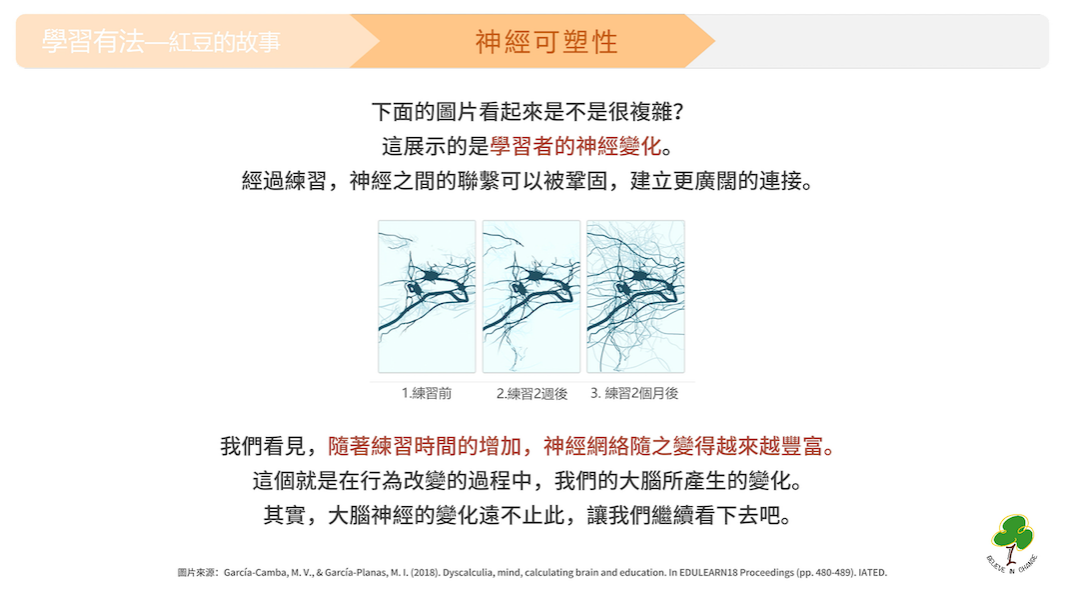
A screenshot demonstrating the neural changes in a learner. The image and text indicate that “Through practice, the connections between neurons can be strengthened, establishing broader connections. We can see that as practice time increases, the neural network becomes progressively more enriched. This is the change that our brains undergo during the process of behavioral change. In fact, the changes in brain neurons go far beyond this. Let's continue to explore.”.

The intervention is a 45-minute web-based course, finally consisting of five elements: (1) an introduction to a proactive mindset, including neuroplasticity, the malleability of intelligence and emotion, and the importance of failure and feedback in the learning process; (2) stories and testimonials during practicum, which emphasize the belief in change; (3) short videos about allegories of developing intelligence, emotion, and failure mindsets; (4) common questions and misconceptions about growth mindsets; and (5) self-persuasive writing exercises in which participants write down their thoughts and suggestions for others about growth mindsets. Figure 1 displays the intervention home page, Figure 2 provides an example of a story emphasizing the positive aspects of failure, and Figure 3 presents the neuroplasticity foundation linked to a growth mindset. Participants in the TAU group will be provided with the We-SMILE after completing the 8-week follow-up.

### Randomization Process

Randomization will be performed via the randomization module of Qualtrics with a 1:1 allocation, whereby students will be allocated to either the We-SMILE group or the TAU group. Students assigned to the We-SMILE group will receive the intervention link immediately, while participants in the TAU group will be provided with the course 8 weeks later. Both groups will be taught by instructors with equivalent qualifications and expertise in the subject matter to ensure consistency in the teaching quality. Concealment can be ensured as participants complete the survey individually and randomization is set after the baseline assessment. The data will be collected at 3 intervals: baseline, 2 weeks after the intervention, and 8 weeks after the intervention; that is, the research team will send the link of follow-up questionnaires to participants at the corresponding time.

### Sample Size and Power Analysis

This study targeted prepracticum social work and counseling students during the period 2024-2025. The sample size is 117, based on the number of trainees. The G*Power is used to determine the target sample size, achieving adequate efficacy to detect mean group differences of small (*d*=0.2), medium (*d*=0.5), and large (*d*=0.8) effect sizes using 2-tailed tests with α=.05. Although previous studies have indicated that the ideal is to detect small effects, the target sample of 117 in this study may reflect the ability to detect moderate to large effects (slightly greater than 0.5) due to limitations on the number of students in the programs [44].

### Participant Eligibility

Undergraduate and postgraduate prepracticum students in social work and counseling programs from universities in Hong Kong and Mainland China are eligible. Inclusion criteria are students who (1) are about to start the practicum soon, (2) can read and write Chinese, and (3) consent to participate. Exclusion criteria are students who (1) do not consent to participate, (2) cannot concentrate for at least 45 minutes to complete the intervention and questionnaires, (3) have a disability or serious physical or mental illness resulting in poor condition, and (4) do not participate in the practicum.

### Measurements

**Figure 4:**
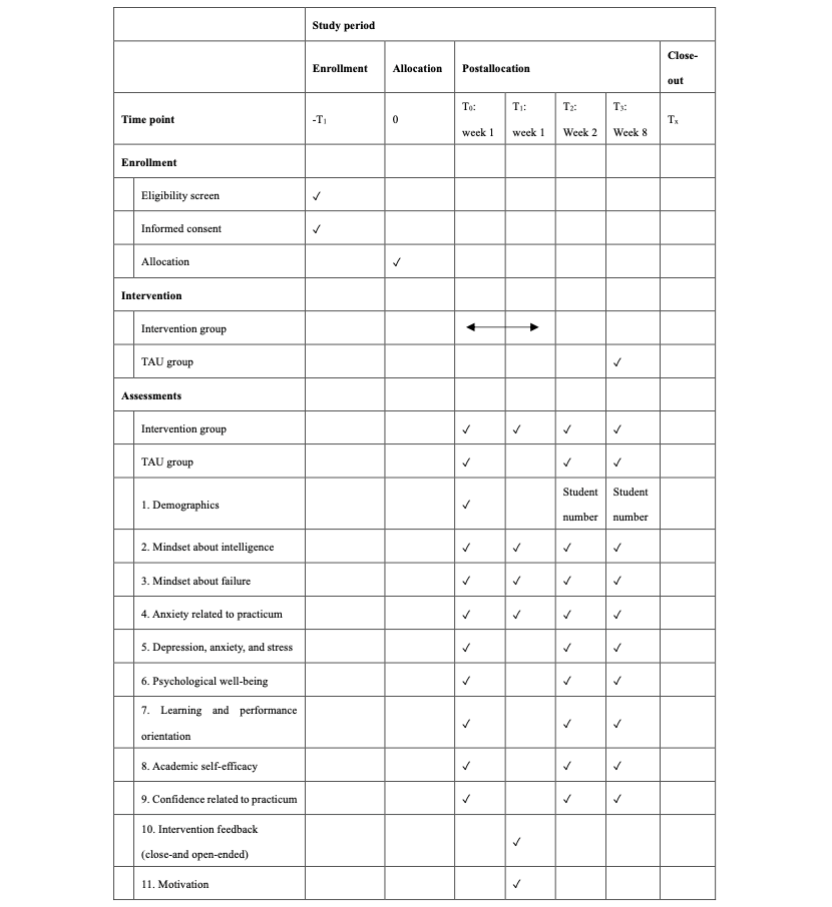
Study periods, arrangements, and measurement schedules. T: time point; TAU: training as usual.

Figure 4 illustrates the specific research arrangements and measurement schedules.

### Demographics

Name, gender, ethnicity, year of birth, year of study, program type, program name, student number, practicum mode, previous social work experience, and other work experience will be collected at baseline. Students’ university numbers will be collected again at 2 follow-ups for matching. Collected students’ names and university numbers are solely for administrative purposes, such as matching pre- and postintervention data and ensuring accurate contact with participants for compensation after this study.

### Fidelity Checking

Mindset about intelligence will be assessed by a 3-item Growth Mindset Scale developed by Dweck et al [45] with high internal reliability across studies, including “you have a certain amount of intelligence, and you can’t really do much to change it,” “your intelligence is something about you that you can’t change very much,” and “you can learn new things, but you can’t really change your basic intelligence.” Each item will be rated from 1=strongly disagree to 6=strongly agree. A higher average score means participants believe less that they can become smarter if they work at it. The reliability was reported in adolescents with Cronbach α of 0.71 [46].

Mindset about failure will be measured by the 6-item Failure Mindset Scale [47,48]. The first 3 items indicate that failure can be a positive motivation (eg, “The effects of failure are positive and should be utilised”), while the last 3 items indicate that failure is a setback (eg, “The effects of failure are negative and should be avoided.”). Each item will be rated from 1 (strongly disagree) to 6 (strongly agree). The Cronbach α was 0.76 [48].

### Primary Outcome Variable

Anxiety related to practicum will be measured by 4 items proposed by Gelman which parallels with the evaluation often used in social work practice [16], including 1 direct measuring item: “level of anxiety about starting the practicum,” and 3 indirect measuring items: “how much your anxiety will interfere with their learning,” “how prepared you are for the practicum” and “how excited you are to participate in the practicum” [16,49]. Each statement will be rated on a 10-point Likert scale, ranging from 1=completely not anxious, very small, completely unprepared, and completely unexcited to 10=extremely anxious, very great, perfectly prepared, and extremely excited [16]. The Cronbach α was 0.70 [50].

### Secondary Outcome Variables

Depression, anxiety, and stress will be assessed by the simplified 12-item Depression Anxiety Stress Scales (DASS-12) [51,52]. The items of DASS-12 were shortlisted from the DASS-21, such as “I found it hard to wind down” [51,52]. Each item will be rated from 0=never to 4=almost always according to the participant’s status over the past week. A higher score indicates a higher level of recognition of one’s recent mental distress symptoms. The Cronbach α was 0.90 [52].

Psychological well-being will be measured using the 7-item Short Warwick-Edinburgh Mental Well-Being Scale [53,54]. One example item is “I’ve been feeling optimistic about the future.” Each item is rated from 1 (none of the time) to 5 (all of the time). Higher scores suggest better mental well-being. The Short Warwick-Edinburgh Mental Well-Being Scale was validated in Hong Kong, and the Cronbach α was 0.89 [55].

Learning and performance orientation will be assessed by 11 adapted items from learning orientation and performance orientation scale [56], with 5 items measuring the learning orientation (eg, “I like to learn new knowledge in practicums”) and 6 items measuring performance orientation (eg, “I like to seek rewards in short term for my efforts”). Each item will be rated on a 5-point Likert scale from 1=strongly disagree to 5=strongly agree. Reliabilities were reported of Cronbach α with 0.65 and 0.56 [56].

Academic self-efficacy will be measured by 5 adapted items from the Bandura Self-Efficacy Scale [57]. Example items are “I believe that as long as I study diligently, I will be able to master practical skills” and “as long as I am diligent, I can master all of the skills learned during the internship.” The items will be scored from 1=strongly disagree to 5=strongly agree and reported a Cronbach α of 0.84 [57].

Confidence related to practicum will be assessed by a 6-item self-developed scale. Items generated from interviews with social work students, such as “I am confident that I can listen to comments from supervisors/colleagues/service users with an open mind” and “I am confident that I can manage my time well and cope with my practicum work and schoolwork at the same time.” Each item needs to be rated from 1=strongly disagree to 5=strongly agree. A higher total score represents a higher level of confidence.

### Intervention Feedback

Intervention feedback contains both close-ended and open-ended questions. The close-ended intervention feedback will be measured using an adapted 12-item 5-point Likert scale based on the Theoretical Framework of Acceptability scale [36]. The items include affective attitude, burden, perceived effectiveness, opportunity costs, and specific acceptability. A sample item is “does this course affect your daily schedule?” The open-ended question invites participants to share their thoughts about the intervention: “Do you have any other suggestions or feedback about this programme?”

Motivation will be measured using 2 self-designed items: “How much do you want to improve your ability to cope with challenges?” and “how much do you want to use what you have learned in the course to cope with challenges in your current or future practicum?” The items will be measured on a 6-point scale from 1=extremely not willing to 6=extremely willing. Higher scores indicate that participants are more motivated to learn from the intervention, apply it to their practice, and are intrinsically more willing to improve their status.

### Pilot Study

The pilot study is initiated at the end of May 2024, before the commencement of the first batch of social work and counseling students’ practicum. An overview of the intervention study is incorporated into the prepracticum briefing to attract participants. Upon obtaining consent, program supervisors will distribute the intervention course link to the students. Alongside standardized questionnaires, participants are invited to offer open-ended feedback on the intervention to facilitate future revisions and enhancements after finishing. Examples of feedback we currently received include “The content and duration can be condensed. The principle about red beans’ story is something I believe everyone understands. It’s better to directly point it out” and “If the voiceover is done by a real person, it would feel more authentic. The sound effects in the stories are very interesting” In the next revised version, the intervention will be shortened and improved with human voice dubbing from a professional actress.

### Follow-Up Plan for Distressed Participants

Follow-up plans have been established to support participants who experienced severe distress during the trial. Research staff have been trained to respond to reports of distress from participants. If severe distress is identified, participants will be referred to appropriate resources, including counseling services and crisis hotlines provided by schools, government agencies, and nongovernmental organizations. Additionally, guidance on seeking professional help is included in the information sheet given to participants. Ensuring the mental health and well-being of participants is always our top priority.

### Analytic Plan

Data analysis will be conducted according to the intention-to-treat (ITT) analysis principle, and missing data will be processed using multiple imputations. Descriptive analyses will be used for the distribution of demographics and all outcome variables. Analysis of covariance will be deployed to compare postintervention outcomes between the intervention and TAU groups, controlling for baseline differences. This approach ensures that the observed effects can be attributed to the intervention rather than preexisting differences, thereby enhancing the validity of the results. Generalized estimating equations will be applied to process data from repeated measures to initiate subgroup analyses and to assess differences in the efficacy of the intervention among participants with different work experiences, emotional states, and mindsets. Because of the cluster randomization, a 2-level analysis will be conducted [58]. Additionally, multilevel regressions will be used to test group effects, time effects, and their interactions on outcome variables. A *P* value of <.05 will be considered statistically significant, and all statistical analyses will be performed using SPSS (version 26; IBM Corp).

### Data Quality Assurance

Respondents can review and change their answers through a “back” button. The investigators will be responsible for the quality control of the data. Data with response times that are too long or too short, as well as expired responses, will be excluded. Completion times from the pilot study will be used to determine thresholds for early or late submissions. The first entry will be kept to avoid duplicate entries. Two attention-checking items are embedded to identify careless responses.

### Protocol Amendments

In general, the research will be conducted following the protocol. The protocol should be seriously and carefully revised and reapproved by the ethics committee of the PI’s university if major modifications are necessary for any reason or if the change will impact the conduct of this study and the interests of the participants. Minor adjustments that do not have significant impacts on this study and participants will be recorded for subsequent supplementation of ethical and trial materials.

### Ethical Considerations

This study has received research ethics approval from the institutional review board of the PI’s university (HSEARS20240512001-01). Students who click the link sent by supervisors and teachers will first read the information sheet and sign the consent form. The participants can decide when to start and withdraw from the intervention. The primary investigators will be responsible for the management of research data. All identifying information of participants will be anonymized during the research process, including data analysis, results reporting, and publication. The data will be generated directly from the web-based survey platform and will be stored at the PI’s university. All database files will be password-protected and can only be accessed by direct researchers. Researchers who have permission to access the data will be appropriately trained to maintain data confidentiality, integrity, and basic data security measures. All data and backups will be maintained till 5 years after the completion of the research. Each eligible participant will be contacted by the research team and receive HK $100 (US $12.87) supermarket vouchers after completing all the questionnaires.

## Results

**Figure 5:**
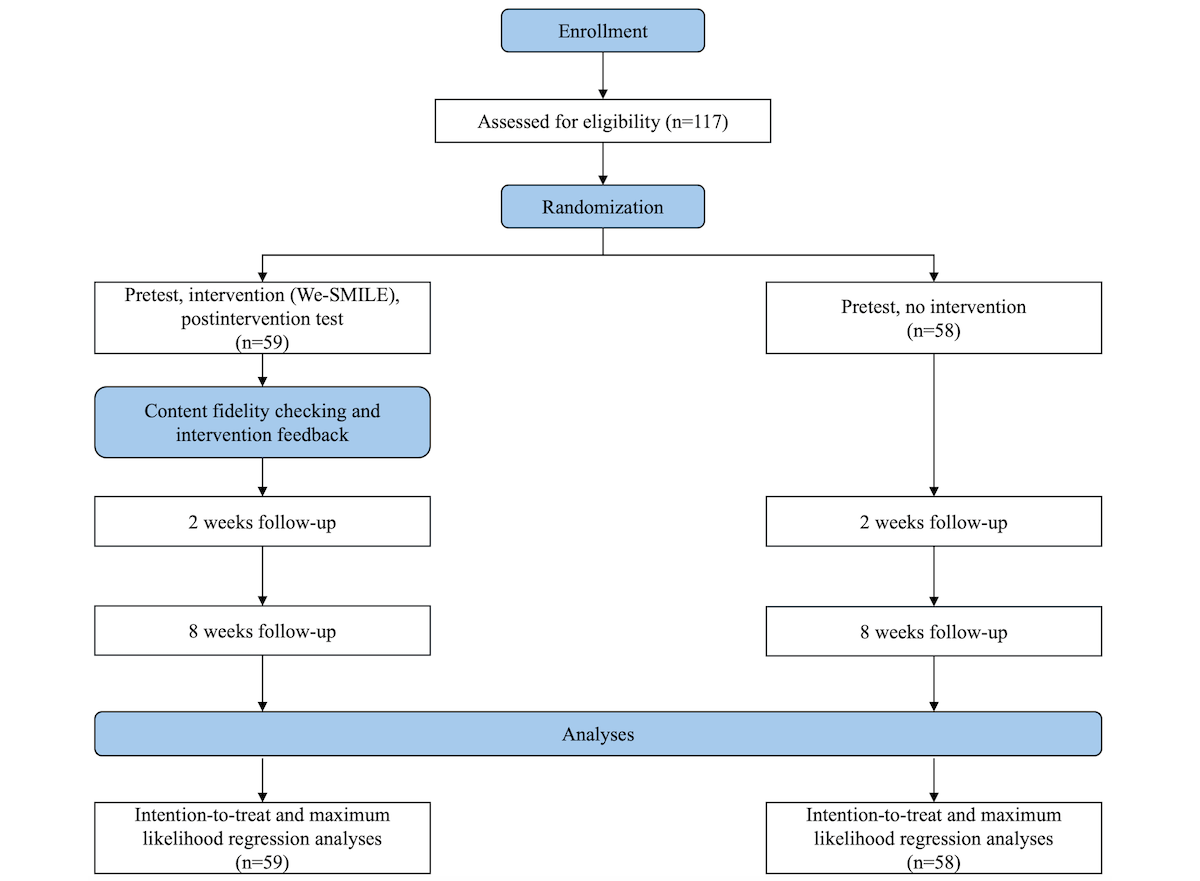
CONSORT diagram. CONSORT: Consolidated Standards of Reporting Trials; We-SMILE: Web-Based Single-Session Intervention of Mindset on Intelligence, Failure, and Emotion.

The project was funded by the Departmental Teaching and Learning Grant of the Department of Applied Social Sciences at PI’s university (grant 8AL1). Recruitment began in May 2024, and data collection is expected to end in April 2025. Data collection is currently underway. The results are scheduled to be released in August 2025. We plan to issue a publication and may also disseminate the results at conferences. The flow of this study is shown in Figure 5.

## Discussion

### Principal Findings

The purpose of this study is to initiate a randomized controlled trial among prepracticum social work and counseling students, assessing the efficacy and acceptability of a digital integrated growth mindset intervention (We-SMILE). It is expected that the We-SMILE group will present lower anxiety related to practicum and more positive secondary outcomes compared to the TAU group. Although this is a single-session, web-based self-help intervention, its efficacy, effectiveness, and sustainability should not be overlooked, as it may continue to serve a useful purpose beyond the project period [44].

We-SMILE is an innovative, convenient, and promising intervention for prepracticum training with a focus on the growth mindset. The We-SMILE will be the key deliverable of the proposed project and has several remarkable advantages. First, We-SMILE is theory-based. Extant interventions with similar core concepts from Western or local practice have been found effective in improving mental health [28,36,40]. Second, patient and public involvement and the coproduction process involving students and fieldwork supervisors ensure We-SMILE to be a tailor-made intervention for practicum trainees. Third, the web-based and nonstigmatizing natures increase accessibility and flexibility for young individuals. Once the efficacy of We-SMILE is established, the integrated mindset prepracticum intervention can help hundreds of students be psychologically prepared for fieldwork training. While providing mental support, it can also supplement practicum education as a readily accessible module to enhance the training outcomes of social work and counseling students. Additionally, this study will provide clear implementation protocols and strategies to increase transparency. The intervention study can be replicated, developed, and referenced as a basis for future research and the design of single-session growth mindset interventions.

### Strengths

Based on previous research indicating the lack of interventions targeting prepracticum training, the proposed study fills this gap by focusing on improving the growth mindset in this particular population. The project will benefit multiple parties. The immediate beneficiaries will be social work and counseling students. The intervention is designed to tune trainees’ mindsets regarding how they perceive the learning process, feedback, failure, and emotion. It could help prevent a high level of anxiety and fear about receiving negative feedback and facilitate students building resilience in fieldwork training. Second, it could benefit fieldwork supervisors. We-SMILE offers a flexible intervention option as a concise psychoeducational tool that seamlessly fits into prepracticum preparation. It does not require an extra workload for supervisors and can easily be distributed for students’ needs and reference. Alternatively, the intervention can be used as a specialized component of the existing prepracticum training or incorporated into classroom teaching. Third, it may benefit the social work and counseling programs as a whole. We-SMILE provides an accessible intervention to assist social work and counseling students in transitioning into future workplaces with lower levels of distress, better self-efficacy, and becoming mentally prepared, which will in return improve the outcome and quality of education programs.

### Limitations

Some limitations in this intervention require further consideration and refinement. First, this study only targets students in social work and counseling programs. The number of participants in each cohort will be limited. We may not be able to recruit a sufficient sample size if practicum trainees are occupied by coursework and internships and become too busy to complete the intervention course. There is a need to expand the scope of implementation by including a larger sample in the future to demonstrate that the intervention could be adapted into training for other education and health care domains. Second, as this study does not differentiate individuals with various levels of anxiety states, the effectiveness in reducing anxiety may not be significant for those students with no or low levels of anxiety, thus, it may affect the overall statistical significance. Therefore, we may conduct subgroup analyses to address this issue.

### Conclusion

This study aims to develop a digital single-session integrated growth mindset intervention (We-SMILE) and to evaluate its efficacy and acceptability using a 2-arm randomized controlled trial among practicum trainees of social work and counseling programs. If proven effective, our study will provide a potential model for the implementation of a brief, low-dose, single-session intervention. In return, We-SMILE may also contribute to the development of accessible, highly adaptable, low-cost, and sustainable interventions in mental health education on a larger scale.

## Appendix

Multimedia Appendix 1 CONSORT-eHEALTH checklist (V1.6.1).

Multimedia Appendix 2 SPIRIT checklist.

## Abbreviations

CONSORT-EHEALTH: Consolidated Standards of Reporting Trials of Electronic and Mobile Health Applications and Online Telehealth
DASS: Depression Anxiety Stress Scales
ITT: intention-to-treat analysis
PC-SMILE: Parent-Child Single-Session Mindset Intervention on Intelligence, Failure, and Emotion
PI: principal investigator
SPIRIT: Standard Protocol Items: Recommendations for Interventional Trials
TAU: training as usual
We-SMILE: Web-Based Single-Session Intervention of Mindset on Intelligence, Failure, and Emotion

## Data Availability

Data generated and analyzed during this study is not publicly available due to privacy and confidentiality issues but can be obtained by contacting the corresponding author upon reasonable request.
